# Standardization and Validation of Antigen Retrieval Methods for Immunofluorescence on Formalin-Fixed, Paraffin-Embedded Biopsies

**DOI:** 10.7759/cureus.105821

**Published:** 2026-03-25

**Authors:** Nipan Das, Biswajit Dey, Rennie O Lakadong, Vandana Raphael

**Affiliations:** 1 Pathology, North Eastern Indira Gandhi Regional Institute of Health and Medical Sciences, Shillong, IND; 2 Allied Health Sciences, Martin Luther Christian University, Shillong, IND

**Keywords:** antibodies, antigen retrieval, formalin-fixed paraffin-embedded tissue, immunofluorescence, proteinase-k

## Abstract

Introduction

Direct immunofluorescence (DIF) performed on fresh-frozen tissue is regarded as the reference standard for detecting immune deposits in renal and skin biopsies. However, limitations, such as inadequate frozen tissue, technical constraints, and the inability to perform retrospective analysis, necessitate alternative approaches. Immunofluorescence on formalin-fixed, paraffin-embedded (FFPE) tissue, combined with effective antigen retrieval methods, has emerged as a potential substitute. This study aimed to standardize and validate different antigen retrieval techniques for immunofluorescence on FFPE tissue and to assess their diagnostic performance in comparison with frozen section immunofluorescence.

Materials and methods

A total of 100 biopsies, comprising 50 native renal biopsies for glomerulonephritis and 50 skin biopsies for immunobullous disorders, were included. The corresponding frozen section immunofluorescence served as the reference standard. FFPE sections were subjected to antigen retrieval using proteinase-K, pronase, trypsin, and dual microwave methods. Fluorescein isothiocyanate (FITC) conjugated antibodies against IgA, IgG, IgM, C3, kappa, and lambda were applied. The staining intensity was assessed using a semi-quantitative scale ranging from 0 to 3+. Accuracy, positive predictive value, negative predictive value, sensitivity, and specificity of each FFPE-based method were calculated in comparison with frozen section immunofluorescence.

Results

Among the antigen retrieval techniques evaluated, proteinase-K demonstrated the highest sensitivity across immunoglobulins and complement components in both renal and skin biopsies. Repeated-measures one-way analysis of variance (ANOVA) showed a statistically significant difference in sensitivity among methods (p = 0.0280), while specificity did not differ significantly, with all methods showing near-perfect specificity.

Conclusion

Immunofluorescence on FFPE tissue using proteinase-K antigen retrieval provides high sensitivity and excellent specificity, closely approximating frozen section results. This method represents a reliable and practical alternative, particularly when frozen tissue is unavailable, and serves as an effective salvage technique in routine diagnostic pathology.

## Introduction

Direct immunofluorescence (DIF) performed on fresh frozen tissue has traditionally been regarded as the reference standard for identifying immune complexes and complement proteins in renal and skin biopsies. Nonetheless, this technique has limitations in clinical application. These drawbacks include the potential for thick tissue sections and diffuse antigen distribution, both of which can hinder accurate interpretation. Additionally, the limited availability of frozen tissue may reduce diagnostic precision, and frozen sections cannot be re-examined retrospectively [1]. In such cases, formalin-fixed, paraffin-embedded (FFPE) tissue immunofluorescence (IF) may serve as a viable alternative. Research has shown that IF on FFPE samples yields results comparable to those on frozen tissue for most immunoglobulins and complement components [2]. However, a major challenge with FFPE tissue is that formalin-induced protein cross-linking can mask antigens, preventing fluorescein isothiocyanate (FITC)-conjugated antibodies from binding effectively [1].

To address this issue, various antigen retrieval techniques have been developed, including the use of enzymes such as proteinase-K, pronase, and trypsin, as well as dual microwave heating. Despite their documented effectiveness, these methods are not yet widely implemented in routine renal and skin biopsy processing [3]. In the current study, proteinase-K, pronase, trypsin, and dual microwave were employed for antigen retrieval. These enzymes help break the protein cross-links caused by formalin fixation, thereby unmasking antigens and enabling FITC-conjugated antibody detection.

This research aimed to evaluate the diagnostic value of paraffin IF using different digestions compared to the conventional method on fresh frozen tissues comprising renal and skin biopsies.

## Materials and methods

FFPE tissue blocks from 100 biopsy cases were included in the study, comprising 50 native renal biopsies for glomerulonephritis and 50 skin biopsies for immunobullous disorders. These cases were retrieved from the archives of the Department of Pathology at the North Eastern Indira Gandhi Regional Institute of Health and Medical Sciences (NEIGRIHMS), Shillong, India. All 100 cases had previously been processed for routine hematoxylin and eosin (H&E) staining and had corresponding DIF studies performed on frozen sections.

For the purpose of conducting IF on FFPE tissue (IF-FFPE), proteinase-K (Sigma-Aldrich, St. Louis, MO; Catalog No. P2308), pronase (*Streptomyces griseus*) (Sigma-Aldrich, St. Louis, MO), and trypsin (2.5% Trypsin, Fisher Scientific, Waltham, MA) were applied to slides prepared from the same tissue blocks originally used for H&E staining. The concentration, incubation period, and temperature for proteinase-K were 1.25 mg/mL in phosphate-buffered saline (PBS), 30 minutes, and 37°C, respectively. For pronase, it was 75 mg/100 mL in Tris buffer for 60 minutes at 37°C; for trypsin, 0.1% in Tris buffer for 30 minutes at 37°C. In the dual microwave method, tissue sections were heated first at 320 W for three minutes to 850 W for 2.5 minutes in a Tris-ethylenediaminetetraacetic acid (EDTA) antigen retrieval solution (0.01 M, pH 8.0). Sections were cooled, washed in PBS, refixed in formaldehyde, and then subjected to a second antigen retrieval in a microwave with sections in Tris-EDTA buffer at 100°C for 10 minutes. They were then used for histopathological evaluation of renal and skin diseases.

Pronase method

We prepared poly-L-lysine (PLL)- coated slides with 3 µm tissue sections and deparaffinized them using standard xylene and ethanol washes. Antigen retrieval was performed using pronase in Tris buffer. We then incubated the slides with fluorescence-conjugated antibodies in a humid chamber. After incubation, the slides were gently rinsed and mounted in aqueous PBS for microscopic evaluation [4].

Trypsin method

We prepared PLL slides with 3 µm tissue sections and deparaffinized them using standard protocols. Trypsin digestion was performed in Tris buffer, and the slides were incubated. After digestion, fluorescent antibodies were applied, followed by gentle rinsing. Finally, the sections were mounted with glycerine for imaging [5].

Dual microwave method

We prepared PLL slides with 1-2 µm tissue sections. Two rounds of microwave antigen retrieval were performed, one before and one after fixation. Fluorescent antibodies were then applied, followed by washing. Finally, the slides were mounted with glycerine [6,7].

Proteinase-K method

We prepared PLL slides with 3 µm tissue sections and deparaffinized them using standard methods. Antigens were unmasked by incubating the slides with proteinase-K in PBS buffer. The slides were then incubated with fluorescent antibodies, rinsed gently, and finally mounted in aqueous PBS [1].

Frozen section method

For the IF-frozen technique, 3-4 µm thick sections were cut using a cryostat and mounted on PLL-coated slides. The sections were air-dried and subsequently washed three times with PBS (pH 7.4). Fluorescein-conjugated antibodies were then applied, and the slides were incubated at 37°C. Following incubation, the sections were again washed three times with PBS and finally mounted using glycerine.

Evaluation of immunofluorescence

Staining was graded and interpreted using a semi-quantitative scale: absent (0), mild (1+), moderate (2+), and strong (3+). The staining intensity of IF-FFPE and IF-frozen methods was evaluated by the fluorescence intensity of FITC-conjugated IgA, IgG, IgM, C3, kappa, and lambda antibodies. The diagnostic performance of IF-FFPE was assessed by determining its sensitivity and specificity in comparison with IF-frozen, which was considered the gold standard technique. All IF-FFPE slides were independently evaluated by a single dermatopathologist who was blinded to the IF-frozen results.

Statistical analysis

Accuracy, positive predictive value, negative predictive value, sensitivity, and specificity of IF on FFPE sections were calculated using IF-frozen as the reference standard. Differences in sensitivity and specificity among the four antigen retrieval methods (pronase, trypsin, dual microwave, and proteinase-K) were evaluated using repeated-measures (RM) one-way analysis of variance (RM one-way ANOVA), with antibody markers (IgA, IgG, IgM, C3, kappa, and lambda) treated as RM. Antibody-specific sensitivity and specificity values constituted the unit of analysis. Because sensitivity and specificity were measured across the same antibody panel for each method, the analysis was performed as a pooled RM comparison to account for within-antibody correlation. Bonferroni-corrected paired post hoc tests were performed where applicable following significant results in the RM one-way ANOVA.

Statistical analyses were performed using Microsoft Excel (Microsoft Corp., Redmond, WA) and GraphPad Prism (GraphPad Software, San Diego, CA). A p-value < 0.05 was considered statistically significant.

## Results

This study evaluated a total of 100 biopsy cases, comprising 50 native renal biopsies for glomerulonephritis and 50 skin biopsies for immunobullous lesions, to compare the performance of IF on FFPE tissue against the reference standard frozen section IF (IF-frozen). The FITC-labelled antibodies evaluated were IgA, IgG, IgM, C3, kappa, and lambda. IgA, IgG, IgM, and C3 were done in all 100 cases, whereas kappa and lambda were done in only 50 cases of renal biopsies.

The intensity of FITC-labelled antibodies was compared between IF-FFPE and IF-frozen sections. Sensitivity and specificity of different FFPE-based IF methods were assessed, using IF-frozen as the reference standard (Tables 1-4).

**Table 1:** Accuracy, PPV, NPV, sensitivity, and specificity for IgA, IgG, IgM, C3, kappa, and lambda by the pronase method of FFPE biopsies CI, confidence interval; FFPE, formalin-fixed, paraffin-embedded; NPV, negative predictive value; PPV, positive predictive value a (+/+): Cases positive in both IF-frozen and IF-FFPE, i.e., true positives b (-/+): Cases negative in IF-frozen but positive in IF-FFPE, i.e., false positives c (+/-): Cases positive in IF-frozen but negative in IF-FFPE, i.e., false negatives d (-/-): Cases negative in both IF-frozen and IF-FFPE, i.e., true negatives

IF-Frozen/IF-FFPE	a (+/+)	b (-/+)	c (+/-)	d (-/-)	No. of Cases	Accuracy	PVV	NPV	Sensitivity (95% CI)	Specificity (95% CI)
Ig A	32	0	3	65	100	97%	100%	95.6%	91.4% (77.6-97)	100% (94.4-100)
IgG	73	0	5	22	100	95%	100%	81.5%	93.6% (85.9-97.2)	100% (85.1-100)
IgM	36	1	4	59	100	95%	97.3%	93.7%	90% (76.9-96)	98.3% (91.1-99.7)
C3	60	0	15	25	100	85%	100%	62.5%	80% (69.6-87.5)	100% (86.7-100)
Kappa	35	0	3	12	50	94%	100%	80%	92.1% (79.2-97.3)	100% (75.8-100)
Lambda	35	0	2	13	50	96%	100%	86.7%	94.6% (82.3-98.5)	100% (77.2-100)

**Table 2:** Accuracy, PPV, NPV, sensitivity, and specificity for IgA, IgG, IgM, C3, kappa, and lambda by the trypsin method of FFPE biopsies CI, confidence interval; FFPE, formalin-fixed, paraffin-embedded; NPV, negative predictive value; PPV, positive predictive value a (+/+): Cases positive in both IF-frozen and IF-FFPE, i.e., true positives b (-/+): Cases negative in IF-frozen but positive in IF-FFPE, i.e., false positives c (+/-): Cases positive in IF-frozen but negative in IF-FFPE, i.e., false negatives d (-/-): Cases negative in both IF-frozen and IF-FFPE, i.e., true negatives

IF-Frozen/IF-FFPE	a (+/+)	b (-/+)	c (+/-)	d (-/-)	No. of Cases	Accuracy	PPV	NPV	Sensitivity (95% CI)	Specificity (95% CI)
Ig A	29	0	5	66	100	95%	100%	93%	85.3% (70.6-93.7)	100% (94.5-100)
IgG	73	0	6	21	100	94%	100%	77.8%	92.4% (84.5-96.5)	100% (84.5-100)
IgM	29	0	10	61	100	90%	100%	85.9%	74.4% (58.5-85.8)	100% (94.1-100)
C3	43	0	32	25	100	68%	100%	43.9%	57.3% (46-67.9)	100% (86.3-100)
Kappa	33	0	5	12	50	90%	100%	70.6%	86.8% (71.1-94.9)	100% (75.8-100)
Lambda	33	0	4	13	50	92%	100%	76.5%	89.2% (75.3-96)	100% (77.2-100)

**Table 3:** Accuracy, PPV, NPV, sensitivity, and specificity for IgA, IgG, IgM, C3, kappa, and lambda by the dual microwave method of FFPE biopsies CI, confidence interval; FFPE, formalin-fixed, paraffin-embedded; NPV, negative predictive value; PPV, positive predictive value a (+/+): Cases positive in both IF-frozen and IF-FFPE, i.e., true positives b (-/+): Cases negative in IF-frozen but positive in IF-FFPE, i.e., false positives c (+/-): Cases positive in IF-frozen but negative in IF-FFPE, i.e., false negatives d (-/-): Cases negative in both IF-frozen and IF-FFPE, i.e., true negatives

IF-Frozen/IF-FFPE	a (+/+)	b (-/+)	c (+/-)	d (-/-)	No. of Cases	Accuracy	PPV	NPV	Sensitivity (95% CI)	Specificity (95% CI)
Ig A	27	0	7	66	100	93%	100%	90.4%	79.4% (63.2-90)	100% (94.5-100)
IgG	68	0	11	21	100	89%	100%	65.6%	86.1% (76.7-92.1)	100% (84.5-100)
IgM	29	0	10	61	100	90%	100%	85.9%	74.4% (58.5-85.8)	100% (94.1-100)
C3	34	0	41	25	100	59%	100%	37.9%	45.3% (34.6-56.5)	100% (86.3-100)
Kappa	30	0	8	12	50	84%	100%	60%	78.9% (62.7-89.7)	100% (75.8-100)
Lambda	31	0	6	13	50	88%	100%	68.4%	83.8% (69.2-92.3)	100% (77.2-100)

**Table 4:** Accuracy, PPV, NPV, sensitivity, and specificity for IgA, IgG, IgM, C3, kappa, and lambda by the proteinase-K method of FFPE biopsies CI, confidence interval; FFPE, formalin-fixed, paraffin-embedded; NPV, negative predictive value; PPV, positive predictive value a (+/+): Cases positive in both IF-frozen and IF-FFPE, i.e., true positives b (-/+): Cases negative in IF-frozen but positive in IF-FFPE, i.e., false positives c (+/-): Cases positive in IF-frozen but negative in IF-FFPE, i.e., false negatives d (-/-): Cases negative in both IF-frozen and IF-FFPE, i.e., true negatives

IF-Frozen/IF-FFPE	a (+/+)	b (-/+)	c (+/-)	d (-/-)	No. of Cases	Accuracy	PPV	NPV	Sensitivity (95% CI)	Specificity (95% CI)
Ig A	34	0	2	64	100	98%	100%	97%	94.4% (81.3-98.6)	100% (94.4-100)
IgG	76	0	4	20	100	96%	100%	83.3%	95% (87.7-98)	100% (83.9-100)
IgM	38	0	3	59	100	97%	100%	95.2%	92.6% (80.1-97.4)	100% (94-100)
C3	61	0	14	25	100	86%	100%	64.1%	81.3% (71-88.4)	100% (86.7-100)
Kappa	35	0	3	12	50	94%	100%	80%	92.1% (79.2-97.3)	100% (75.8-100)
Lambda	35	0	2	13	50	96%	100%	86.7%	94.6% (82.3-98.5)	100% (77.2-100)

**Figure 1:**
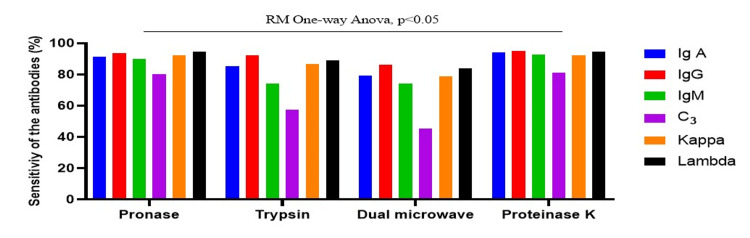
Sensitivity across antigen retrieval methods of IF-FFPE analyzed by pooled RM one-way ANOVA with antibody-specific sensitivity as the unit of analysis ANOVA, analysis of variance; IF-FFPE, immunofluorescence on formalin-fixed, paraffin-embedded; RM, repeated measures

**Figure 2:**
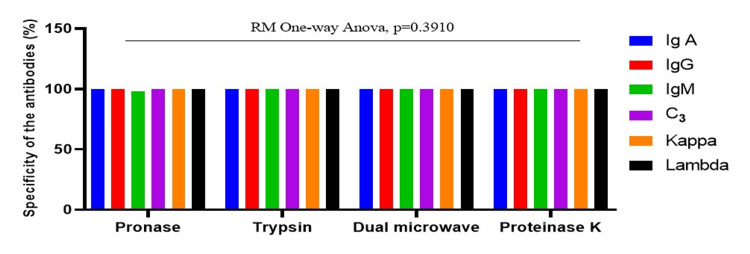
Specificity across antigen retrieval methods of IF-FFPE analyzed by pooled RM one-way ANOVA with antibody-specific specificity as the unit of analysis ANOVA, analysis of variance; IF-FFPE, immunofluorescence on formalin-fixed, paraffin-embedded; RM, repeated measures

An RM one-way ANOVA revealed significant differences in sensitivity among antigen retrieval methods (F = 12.63, p = 0.0280) (Figure 1). Proteinase-K exhibited the highest sensitivity across all markers (IgA, IgG, IgM, C3) and light chains (kappa, lambda) in the biopsies. In contrast, specificity did not significantly differ among methods (p = 0.3910), with all achieving 100% specificity (Figure 2).

Bonferroni-corrected paired post hoc tests were performed for sensitivity, where the overall ANOVA was significant, while post hoc testing was not conducted for specificity due to the absence of significant differences. Post hoc pairwise comparisons demonstrated that proteinase-K showed significantly higher sensitivity compared with the dual microwave method (adjusted p = 0.049), while no other pairwise differences between antigen retrieval methods reached statistical significance. Overall, proteinase-K showed the highest sensitivity among the evaluated methods, suggesting it may be a preferable approach for IF-FFPE analysis in tissue biopsies.

## Discussion

The present study assessed the sensitivity and specificity of four IF-FFPE antigen retrieval methods, proteinase-K, pronase, trypsin, and dual microwave, in both skin and kidney biopsies. Fifty cases each of glomerulonephritis and immunobullous skin lesions were included. These were selected to represent two major diagnostic settings in which DIF is routinely used, allowing evaluation of the performance of IF-FFPE in both renal and skin specimens [8-10].

Among the antigen retrieval methods evaluated, proteinase-K demonstrated the highest sensitivity across immunoglobulins and complement components, with comparable specificity across all methods, indicating its potential utility for IF-FFPE analysis. The proteinase-K method demonstrated the highest sensitivity for IgA (94.4%), IgG (95%), IgM (92.6%), and C3 (81.3%). However, for kappa and lambda antibodies, the proteinase-K method showed sensitivities of 92.1% and 98.6%, which were similar to those of the pronase method. A RM one-way ANOVA revealed a statistically significant difference among the antigen retrieval methods in sensitivity (F = 12.63, p = 0.0280), suggesting variability in their effectiveness for IF-FFPE analysis across kidney and skin biopsies. Post hoc pairwise analysis demonstrated that proteinase-K exhibited significantly greater sensitivity than the dual microwave method (adjusted p = 0.049), whereas no other pairwise comparisons between antigen retrieval methods showed statistically significant differences. Notably, proteinase-K consistently yielded the highest sensitivity among the evaluated methods, indicating that it may represent a more effective approach for IF-FFPE analysis in biopsy specimens.

In contrast, an RM one-way ANOVA for specificity did not reveal significant differences among the antigen retrieval methods (p > 0.05), confirming that all methods performed equally well in terms of specificity. Proteinase-K, trypsin, and dual microwave each achieved 100% specificity for IgA, IgG, and C3, as well as for kappa and lambda light chains in biopsies. The pronase method also showed 100% specificity for IgA, IgG, C3, kappa, and lambda, except for IgM (98.3%). This perfect specificity suggests that all methods are highly reliable in accurately identifying negative cases and minimizing false positives.

Analysis of studies using DIF on paraffin-embedded renal biopsies showed that proteinase-K provided optimal antigen retrieval for immunoglobulins and C3, with a good balance of sensitivity and specificity compared to frozen sections [4,11]. However, pronase demonstrated superior performance in certain cases with heavy immunoglobulin deposition [4,11].

Similarly, other studies have validated proteinase-K for antigen retrieval in FFPE skin biopsies, demonstrating reliable immunoglobulins and complement detection across dermatological pathologies [12,13].

Regarding the intensity of immunoglobulins and complement components, most studies report comparable results between techniques. Comparisons of cases stained using both IF-paraffin and IF-frozen methods demonstrate largely similar staining intensities for diagnostic immunoglobulins or complements, with only minor differences noted [14].

A major strength of this study was the systematic evaluation of multiple antigen retrieval methods on FFPE tissue using frozen section IF as the reference standard. Another key strength of the study was the inclusion of both kidney and skin biopsies, enabling evaluation of antigen retrieval methods across different tissue types. Thus, the study demonstrated the value of IF-FFPE, particularly the proteinase-K method, as a reliable salvage technique.

The study has a few limitations. First, the sample size, although inclusive of both renal and skin biopsies, remains relatively modest and may limit the generalizability of the findings across all disease subtypes. Second, antigen retrieval conditions like enzyme concentration, incubation time, and temperature were standardized for this study, but minor variations in laboratory practice could influence reproducibility and staining intensity in routine settings. Older blocks may affect the results due to degradation processes over time. Additionally, interpretation of IF intensity remains semi-quantitative and observer-dependent, despite blinding, which may affect consistency across observers.

## Conclusions

IF performed on frozen tissue remains the reference standard for diagnosing immune-mediated diseases; however, practical limitations necessitate reliable alternatives. This study shows that IF on FFPE tissue using the proteinase-K antigen retrieval method provides high sensitivity and excellent specificity for detecting immunoglobulins and complement components in FFPE biopsies. Larger studies are necessary to validate findings and refine antigen retrieval techniques. Proteinase-K is a reliable method for detecting immune deposits in FFPE tissues, enhancing diagnostic capabilities in routine pathology practice.
